# Zilebesiran—Small Interfering RNAs as Prospective New Drug in the Treatment of Hypertension

**DOI:** 10.3390/jcm14207365

**Published:** 2025-10-17

**Authors:** Sylwia Dudzicz-Gojowy, Marcin Adamczak

**Affiliations:** Department of Nephrology, Transplantation and Internal Medicine, Medical University of Silesia, 40-027 Katowice, Poland; sdudzicz@sum.edu.pl

**Keywords:** zilebesiran, small interfering RNA, arterial hypertension, renin–angiotensin–aldosterone system, ACEIs and ARBs

## Abstract

The inhibition of renin–angiotensin–aldosterone system (RAAS) activity is one of the key mechanisms in the treatment of arterial hypertension. Non-adherence to therapeutic recommendations is considered to be the main cause of failure to achieve therapeutic goals in patients with arterial hypertension. Zilebesiran is the first antihypertensive drug using expression genes modified by siRNA action. The mechanism of action is based on silencing the angiotensinogen gene by activating the RNA-induced silencing complex (RISC). The decreased production of angiotensinogen and storage of siRNA in hepatocyte endosomes makes the drug’s effect prolonged; it may last up to several months after drug administration. In hypertensive patients, a long-term reduction in blood pressure by more than 10 mmHg compared to placebo has been observed after a single dose of zilebesiran. Despite the promising results of the previous studies, further observations are still necessary regarding side effects and long-term effectiveness, as well as the possibility of developing resistance to treatment.

## 1. Introduction

**Figure 1:**
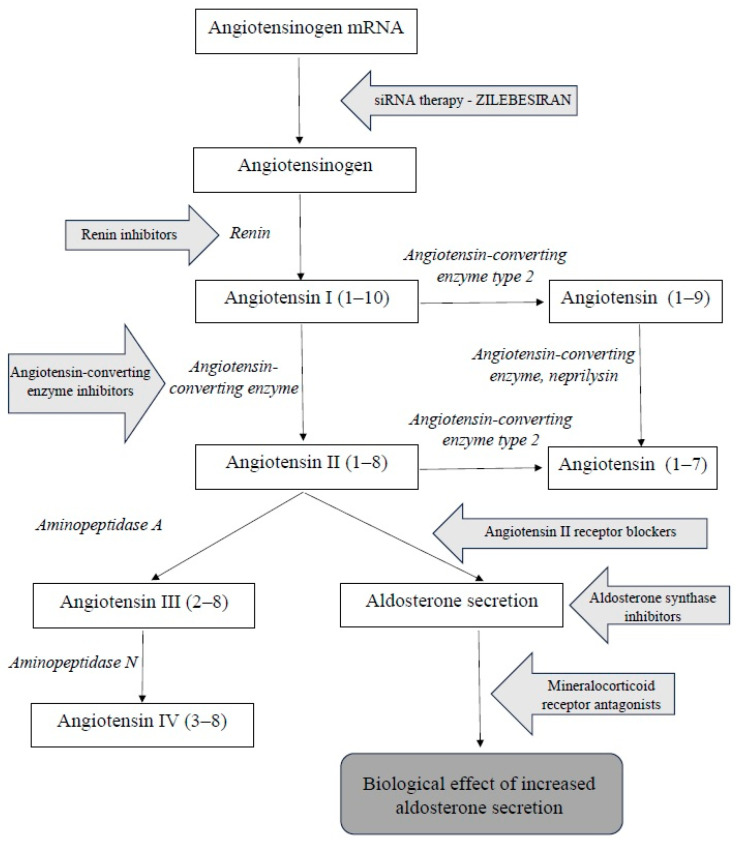
The renin–angiotensin–aldosterone system pathway (siRNA—ang. small interfering RNA).

The renin–angiotensin–aldosterone system (RAAS) is a complex hormonal cascade essential for the regulation of blood pressure and fluid balance. The substrate for this system is angiotensinogen. Renin–angiotensin–aldosterone system consists of two parts: systemic and tissue-based. The systemic RAAS primarily depends on the activity of the juxtaglomerular apparatus in the kidneys. Tissue RAASs act locally and are present in various tissues, including, among others, the central nervous system, kidneys, heart, vascular endothelium, adipose tissue, adrenal glands, and placenta. The systemic RAAS cascade is initiated by the increased production of the proteolytic enzyme renin by the juxtaglomerular cells of the afferent arteriole of the nephron. This occurs in response to a decreased sodium tubular concentration, detected by the macula densa; the activation of the sympathetic nervous system; and reduced renal perfusion pressure. The substrate of RAAS cascade is the protein angiotensinogen, which is produced mainly by hepatocytes. Renin cleaves angiotensinogen into the decapeptide angiotensin I (angiotensin 1–10), which itself has minimal biological activity but serves as a direct precursor to angiotensin II and an indirect precursor to other angiotensin peptides. The conversion of angiotensin I into the biologically active octapeptide angiotensin II (angiotensin 1–8) is catalyzed by angiotensin-converting enzyme (ACE), primarily in the lungs. Angiotensin II exerts a wide range of physiological effects by binding to type 1 (AT1) and type 2 (AT2) receptors. The activation of AT1 and AT2 receptors has opposing effects. The main effect of angiotensin II largely occurs due to the stimulation of AT1 receptors. They are located in blood vessels (mainly in smooth muscle cells and in endothelium), kidneys, the adrenal cortex, lungs, and liver. After binding to angiotensin II, the stimulation of AT1 receptors causes vasoconstriction and an increase in blood pressure by increasing sodium reabsorption, thus stimulating aldosterone and vasopressin secretion. These processes collectively lead to increased circulating blood volume, improved vascular bed filling, and elevated blood pressure [1,2,3,4,5,6]. The excessive stimulation of these receptors can lead to vascular endothelial dysfunction, vascular remodeling, fibrosis, hypertrophy, and inflammation, which are adverse effects of action mainly seen in the heart and blood vessels [7,8,9]. In the pharmacotherapy of hypertension, AT1 receptors are a target for AT1 receptor antagonists (ARBs), thus blocking the effect of angiotensin II and lowering blood pressure. AT2 receptor stimulation has an effect opposite to the activation of AT1 receptors and is sometimes referred to as the protective arm of the RAAS. AT2 receptor stimulation causes vasodilation mediated by nitric oxide and prostaglandins, increased natriuresis, and reduced blood pressure. Additional positive effects include antiproliferative and proapoptotic effects as well as the inhibition of inflammation, oxidative stress, and fibrosis in the heart, kidneys, and vessels. Thanks to this, the activation of AT2 can counteract the harmful effects of excessive AT1 receptor activation [10] (Figure 1).

The inhibition of the renin–angiotensin–aldosterone system (RAAS) is one of the key mechanisms in the treatment of hypertension. Due to the multi-step activation of the RAAS, several drug classes have been developed that target different components of this system. The first group comprises direct renin inhibitors, which reduce renin activity and thereby inhibit the conversion of angiotensinogen into angiotensin I. This leads to a reduction in RAAS activation and a decrease in angiotensin I and II concentrations, without increasing plasma renin activity. A widely used group of drugs is angiotensin-converting enzyme inhibitors (ACEIs), which block the conversion of angiotensin I into angiotensin II by inhibiting the activity of the converting enzyme. Another class includes angiotensin II receptor blockers (ARBs), which prevent the action of angiotensin II by blocking the AT1 receptors. Aldosterone synthase inhibitors (ASIs) inhibit aldosterone production in adrenal glands. Mineralocorticoid receptor antagonists (MRAs) block the effects of aldosterone in the distal renal tubules.

Combination therapy, involving the dual blockade of the RAAS with ACEIs and ARBs, is currently not advised.

The inhibition of the RAAS yields numerous therapeutic benefits. By reducing the production of angiotensin II (via ACEIs) or blocking its effects at the AT1 receptor (via ARBs), vasoconstriction is diminished, vasodilation is promoted, peripheral resistance is lowered, and arterial remodeling is reduced. Furthermore, RAAS-inhibiting drugs possess cardioprotective and nephroprotective properties by preventing myocardial and vascular hypertrophy and fibrosis, slowing the progression of chronic kidney disease and reducing the risk of cardiovascular events.

The European Society of Hypertension (ESH) Clinical Practice Guidelines, published in 2023, and The Polish Society of Hypertension Guidelines, published in 2024, recommend the use, among others, of angiotensin-converting enzyme inhibitors (ACEIs) and angiotensin receptor blockers (ARBs) as basic antihypertensive therapy. According to these guidelines, the dual combination of ACEIs or ARBs and CBBs or diuretics is recommended as the first step. This allows us to achieve proper blood pressure control in about 60% of patients with hypertension. Due to the limited effectiveness of monotherapy, it is recommended to start hypertension therapy with combined treatment, preferably in the form of fixed-dose combinations. After 6–8 weeks from the start of two-drug therapy, its effectiveness should be assessed. If the target BP values are not achieved, it is recommended to use a triple combination consisting of ACEIs or ARBs and CBBs and diuretics. This allows us to achieve proper control of blood pressure in about 90% of patients with hypertension. It is preferred to use combined preparations in a single daily dose administered in the morning hours, which allows us to reduce the non-adherence effect in hypertensive patients. In the remaining patients, it is recommended to extend pharmacotherapy with other drugs or diagnostic methods for secondary hypertension [11,12].

According to the international guidelines like KDIGO 2021 Clinical Practice Guideline on the Management of Blood Pressure (BP) in Chronic Kidney Disease (CKD) [13] and national guidelines like The Polish Society of Nephrology Clinical Practice Position Statements of Pharmacological Nephroprotection in Chronic Kidney Disease Patients with [14] or without Type 2 Diabetes Mellitus [15], it is recommended to start treatment with a dual or triple therapy, using a combination of a thiazide diuretic and/or ACEI/ARB and/or CCB. ACEIs and ARBs are recommended for the initial treatment of hypertension in all patients with chronic kidney disease, regardless of the degree of albuminuria and concomitant diabetes. In patients with CKD, ACEIs and ARBs, apart from their significant antihypertensive role, also play a key role as nephroprotective drugs with a proteinuria-reducing effect [14,15]. ACEIs or ARBs should be used at the maximum tolerated doses. Results of meta-analysis completed by Jafar et al. suggest that both ACEIs and ARBs reduced the risk of renal failure and serious cardiovascular events. In addition, ACEIs reduced the probability of all-cause mortality compared with the control group [16]. During ACEI or ARB therapy, the serum potassium concentration may increase due to the suppression of aldosterone production and eGFR may decrease due to the dilatation of the efferent glomerular arterioles, which leads to decreased intraglomerular filtration pressure. Because of hyperkalemia risk, the measurement of the serum potassium concentration is recommended before and 1–2 weeks after the initiation of ACEI/ARB therapy. eGFR reduction usually occurs within the first 2 weeks of the initiation of therapy. Therefore, patients should be monitored for symptomatic hypotension, hyperkalemia, and serum creatinine concentrations within 2–4 weeks after initiation or dose changes. An eGFR reduction (or increase in serum creatinine concentrations) of <30% from baseline does not require a dose reduction or the discontinuation of ACEI/ARB therapy. In a patient with CKD who develops hyperkalemia after receiving ACEIs/ARBs, it is advisable to implement measures to lower serum potassium concentration. Examples include dietary intake potassium reduction; therapy with preparations that reduce potassium absorption in the gastrointestinal tract and are registered for chronic use (calcium patiromer or sodium zirconium cyclosilicate); thiazide, thiazide-like, or loop diuretics; and the treatment of metabolic acidosis, allowing the continuation of ACEI/ARB therapy at the recommended dose. In patients with CKD and eGFR < 30 mL/min per 1.73 m^2^, the close monitoring of serum potassium concentrations is required during ACEI/ARB therapy. In the case of hypotension in patients with additional indications for ACEI/ARB treatment (albuminuria, heart failure), before reducing the dose or discontinuing treatment with these drugs, it is advisable to consider reducing the dose or discontinuing antihypertensive drugs from other groups used by the patient [13,14,15].

In the light of the extensive benefits of blocking the renin–angiotensin–aldosterone system (RAAS) at various levels, the continued search for new therapeutic targets within this system appears justified.

The main groups of drugs currently used in the treatment of hypertension (ACEIs, ARBs and MRAs) have nephroprotective and cardioprotective effects. In the case of siRNA therapy, zilebesiran still requires further research to assess its nephroprotective and cardioprotective effects. The KARDIA-3 study is currently underway, aimed, among other things, at expanding our knowledge in this area.

During the long-term use of ACEIs and ARBs, the so-called escape phenomenon, or an increase in aldosterone concentration despite the blockade of ACE and the AT1 receptor, can be observed. This leads to the partial or complete loss of effectiveness of treatment with these drug classes. Such an effect might not be present during zilebesiran treatment.

Hyperkalemia is one of the most common side effects of MRAs. Hyperkalemia with zilebesiran was relatively rare and moderate. The possible lack of escape phenomenon and low hyperkalemia risk during zilebesiran therapy support the use of this drug and may make it an attractive alternative to current treatment.

Non-adherence to therapeutic recommendations is considered to be the main cause of failure to achieve therapeutic goals of hypertension treatments. It is most often observed both in young and elderly patients. Non-adherence to therapeutic recommendations in the treatment of arterial hypertension results in the more frequent occurrence of cardiovascular events in the future. In metanalysis published by Lee et al., among 123,390 participants aged 20 to 44, divided according to adherence to recommendations during a 10-year observation period, a nearly 30% higher incidence of cardiovascular diseases was found in non-adherence patients [17]. In another meta-analysis of 23.8 million American adults with hypertension, Chang et al. showed that the rate of non-adherence among this group of patients was 31% and was significantly higher in young adults (aged 18–34 years–58.1%; aged 65–74 years–24.4%) [18]. As can be seen, non-adherence among patients with hypertension is a significant problem. Hypertension is a global problem, especially in developed and developing countries, due to significant lifestyle changes in recent decades. Given the prevalence of hypertension in the global population and the multitude of complications related to this disease, it is crucial to find appropriate pharmacotherapeutic strategies to support patients in the therapeutic process. The use of siRNA drugs, including zilebesiran, may be a breakthrough. To minimize the risk of non-compliance, it is also worth paying attention to the method of administration and the dosage of the drugs used. Non-compliance with the recommendations increases with the number of drugs used and with the frequency of their use during the day. The most optimal solution in this situation is to use combined preparations that will reduce the number of tablets taken once a day, preferably in the morning [19,20]. In light of the above information, very long-acting drugs such as zilebesiran may provide new hope for reducing non-adherence among patients. A single administration of this drug in a six-month period allows better blood pressure control. First of all, it eliminates the daily need to take the drug, which should significantly reduce non-adherence. It may also allow for a reduction in the number of additional antihypertensive drugs, which will be more patient-friendly and will also improve their compliance with the recommendations. In addition, the drug could be used in the form of a therapeutic program in which the patient receives electronic reminders (email, text messages) informing about the approaching date of the next dose and the need to report to their attending physician. In the long term, this form of treatment may reduce the occurrence of cardiovascular complications in the population, reduce the need for hospitalization, and at the same time reduce the global costs of treatment.

## 2. Zilebesiran—Mechanism of Action

Zilebesiran is the first antihypertensive drug using an expression gene modified by siRNA action. The mechanism of action is based on silencing the angiotensinogen gene by activating the RNA-induced silencing complex (RISC). This is a protein complex with endonuclease activity that takes part in the process of silencing gene expression with the participation of double-stranded RNA. The main goal of using this drug is to achieve a reduction in the serum concentration of hepatic angiotensinogen mRNA and thus reduce the production of angiotensinogen. Zilebesiran consists of a short, double-stranded RNA sequence composed of approx. 20 pairs of nucleosomes are connected to N-acetylgalactosamine (GalNAc). The mentioned RNA sequence consists of two strands, sense and antisense, with the latter being the so-called guide strand. Zilebesiran binds specifically to the hepatocyte through receptors for N-acetylgalactosamine. The combination of siRNA with GalNAc allows for high affinity for hepatocytes to be obtained because the mentioned receptors are only located on these cells of the human body. After binding to the N-acetylgalactosamine receptor, the drug is transported into the cell by clathrin-mediated endocytosis and is stored in endosomes. Clathrin-mediated endocytosis is a highly specific process based on the binding of a specific ligand to a receptor, enabling the transmembrane transport of large molecules. It occurs in several steps, involving accessory proteins such as the FCHO protein, responsible for endosomal vesicle formation; the AP2 adaptor protein, which leads to clathrin polymerization; and BAR domain proteins and dynamin, responsible for detaching the endosomal vesicle from the plasma membrane. After endosomal vesicle formation, the clathrin polymer network is disassembled by the HSC70 protein and auxilin. Clathrin molecules can be reused in the endocytosis process. Initially, early endosomes are produced, which eventually transform into late endosomes. As the pH inside the endosome decreases, the endosomal membrane ruptures, releasing the stored substance. After release from the endosome, the sense strand of siRNA is degraded. The antisense strand of siRNA (guide strand) binds to the cytoplasm of the hepatocyte with the RNA-induced silencing complex (RISC), creating an active RISC. Active RISC finds in the cytoplasm angiotensinogen mRNA molecules complementary to the single-stranded siRNA molecule and degrades them. As a result of this process, the expression of the angiotensinogen gene is inhibited, which prevents its further translation and the appearance of protein products [21,22,23]. The active RISC with the guide strand is recycled, so the same complex can degrade many angiotensinogen mRNAs. This mechanism of action and the storage of siRNA in hepatocyte endosomes makes the drug’s effect prolonged and may last up to several months after drug administration [24].

**Figure 2:**
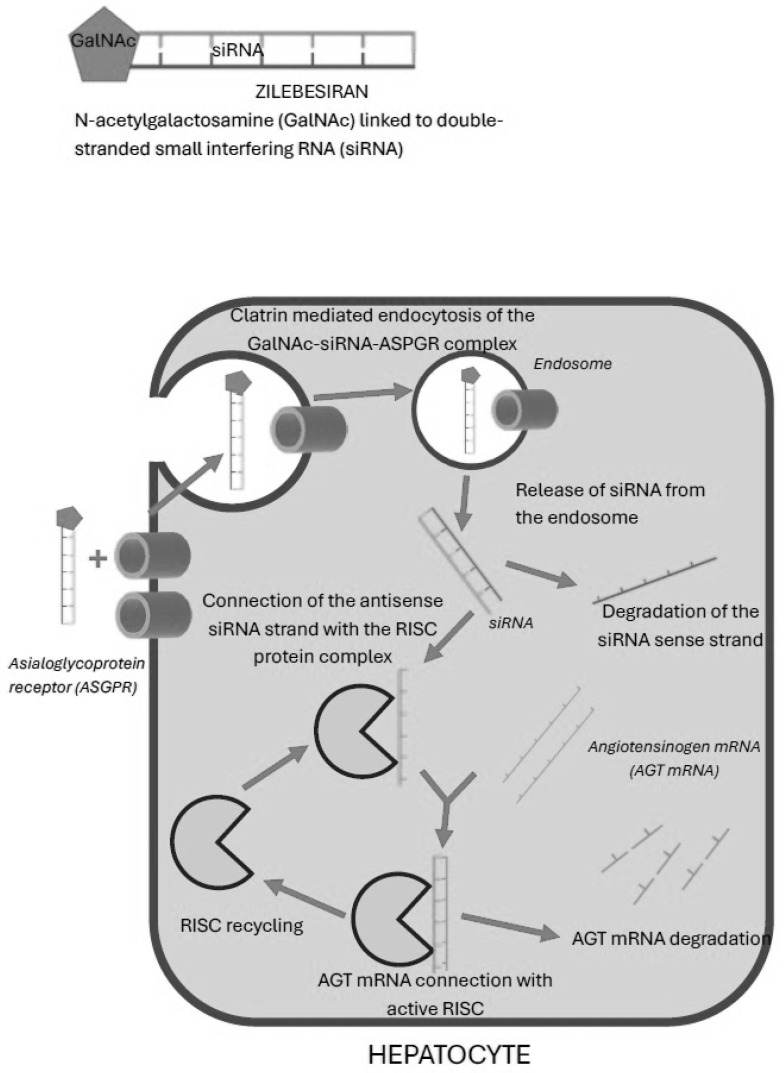
Zilebesiran—mechanism of action.

In summary, the action of zilebesiran is organ-specific because the asialoglycoprotein receptor which binds N-acetylgalactosamine occurs only in the cell membrane of hepatocytes. The complete specificity of zilebesiran’s action, inhibiting the expression of angiotensinogen only, results from the unique sequence of siRNA bases complementary, only to angiotensinogen mRNA. The long-lasting effect of zilebesiran is caused by the slow transfer of siRNA from endosomes and the possibility of the multiple degradation of angiotensinogen mRNA by the activated RISC—single-stranded antisense RNA [25] (Figure 2).

## 3. Zilebesiran—Animal Study Results

Animal studies have provided detailed information on the mechanism of action and the efficacy of angiotensinogen siRNA in the treatment of hypertension. Uijl et al. analyzed the effect of AGT siRNA on angiotensinogen concentrations and its efficacy in the treatment of hypertension [26]. The study involved 12-week-old spontaneously hypertensive male rats fed a standard high-sodium diet. Rats were divided into groups and received valsartan, captopril, captopril + valsartan, AGT siRNA, or valsartan + siRNA for 4 weeks. AGT siRNA was administered by subcutaneous injection once every two weeks at a dose of 10 mg/kg. AGT siRNA reduced serum angiotensinogen concentrations by 98%. Mean arterial pressure was reduced by approximately 14 mmHg after AGT siRNA use and approximately 10 mm Hg after valsartan use, while the combination of AGT siRNA with valsartan reduced mean arterial pressure by up to approximately 70 mmHg. Additionally, the study analyzed the effects of the drugs on cardiac hypertrophy and renal function. Myocardial mass showed a strong positive correlation with mean arterial pressure (r = 0.84; *p* ≤ 0.0001). The improvement in myocardial mass, measured as the ratio of myocardial mass to tibia length, was greatest when siRNA AGT was combined with valsartan, followed by dual RAAS blockade (captopril + valsartan). SiRNA AGT monotherapy resulted in a moderate improvement in cardiac hypertrophy, similar to what occurred with captopril. No cases of acute renal failure were observed in any group of rats studied. GFR at baseline was 1.3 ± 0.1 mL/min per 100 g body weight and remained stable throughout the 4-week treatment period in all groups [26]. Bovée et al. evaluated the efficacy of AGT siRNA in an animal model of chronic kidney disease [27]. Six-week-old male Sprague–Dawley rats fed a standard sodium chloride diet were used. Chronic kidney disease was induced by right nephrectomy and partial left nephrectomy performed 5 weeks before the study. Rats were assigned to placebo, AGT siRNA, AGT siRNA plus losartan, losartan, and losartan plus captopril groups, respectively. Baseline mean arterial pressure was 160 ± 6 mm Hg. During the subsequent 4 weeks of observation, MAP increased to 174 ± 5 mm Hg in the placebo group. AGT siRNA treatment prevented this increase (*p* < 0.05 compared with placebo). Furthermore, AGT siRNA reduced serum angiotensinogen levels by more than 95% [27]. Cruz-López et al. evaluated the efficacy of AGT siRNA in an animal model of diabetes [28]. They studied 10-week-old male heterozygous Ren2 rats fed a standard diet. Diabetes was induced by the intraperitoneal administration of streptozotocin. Rats were divided into groups receiving placebo, captopril, valsartan, AGT siRNA (30 mg/kg every 2 weeks), captopril/valsartan, or AGT siRNA/valsartan. Treatment with AGT siRNA, valsartan, or captopril, and dual therapy with captopril/valsartan, resulted in similar reductions in mean arterial pressure. The greatest reduction in mean arterial pressure was observed after dual therapy with siRNA AGT and valsartan. The reduction in serum angiotensinogen concentration after the use of siRNA AGT was approximately 99% [28].

## 4. Zilebesiran—Clinical Trial Results

A multicenter phase 1 clinical trial evaluated the safety, pharmacokinetic and pharmacodynamic properties, and antihypertensive efficacy of zilebesiran. The study was conducted at four sites in the UK and ran from 30 May 2019 to 26 January 2022. The study consisted of four parts, which were double-blind, randomized, placebo-controlled studies. They evaluated the efficacy of a single ascending dose, a single fixed dose in the presence of a low- and high-salt diet, and a single fixed dose of zilebesiran with concomitant irbesartan. The study included patients aged 18 to 65 years with treated or untreated hypertension who had a mean systolic blood pressure of 130 mm Hg or higher based on 24 h ambulatory blood pressure measurement after discontinuing antihypertensive medication for at least 2 weeks. Hypertensive patients were randomly assigned 2:1 to zilebesiran or placebo. In the first part of the study, they received a single ascending dose of zilebesiran (10, 25, 50, 100, 200, 400, or 800 mg) administered subcutaneously or placebo and were followed for 24 weeks. The second part assessed the effect of an 800 mg dose of zilebesiran on blood pressure in the presence of a low- or high-salt diet and then the effect of this dose given concomitantly with irbesartan. A total of 107 patients were included in the study. Patients receiving zilebesiran experienced dose-related decreases in serum angiotensinogen using approximately 97% concentrations (r = −0.56 at week 8; 95% confidence interval, −0.69 to −0.39). Zilebesiran’s doses of 100 mg or more reduced serum angiotensinogen concentrations by more than 90% from weeks 3 to 12 of the study. In patients receiving 800 mg of zilebesiran, the reduction in serum angiotensinogen concentrations by more than 90% was maintained through week 24 of follow-up. In addition, doses of zilebesiran of 200 mg or more caused small reductions in plasma renin activity, aldosterone, angiotensin I, and angiotensin II serum concentrations. A negative correlation was observed between zilebesiran dose and reduction in mean 24 h systolic blood pressure (r = −0.41 at week 8; 95% CI, −0.58 to −0.21). Single doses of zilebesiran ≥ 200 mg were associated with decreases in systolic blood pressure (>10 mm Hg) and diastolic blood pressure (>5 mm Hg) through week 8; these changes were consistent across the circadian cycle and were maintained through 24 weeks. The reduction in systolic blood pressure correlated with the degree of reduction in serum angiotensinogen levels (r = 0.52; 95% CI, 0.42 to 0.61). There were no cases of hypotension, hyperkalemia, or worsening renal function requiring medical intervention [29].

**Figure 3:**
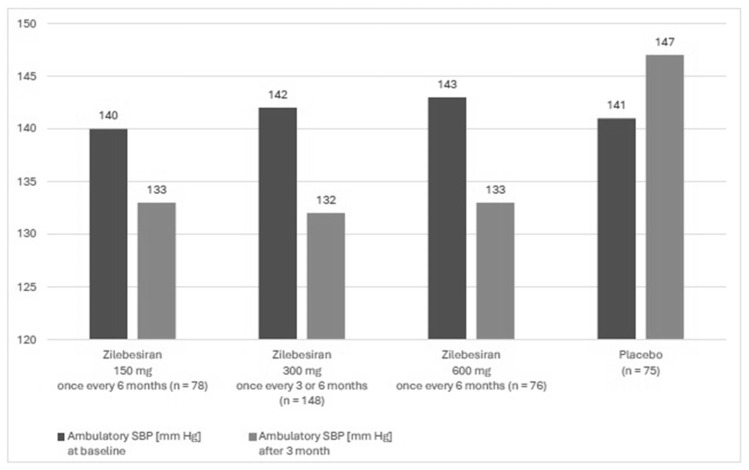
Change in ambulatory SBP depending on the time of administration and dose of zilebesiran compared to placebo (based on Bakris et al. [25]).

A phase 2, randomized, double-blind, dose-ranging, and placebo-controlled study KARDIA-1 assessed the efficacy and safety of zilebesiran in the treatment of hypertension. The study mainly aimed to evaluate different dosing regimens of zilebesiran. The study lasted from July 2021 to June 2023 and it was conducted in 78 centers in four countries (USA, Canada, Great Britain and Ukraine). Patients aged 18 to 75 years with moderate hypertension, defined as mean daily ambulatory systolic blood pressure (SBP) between 135 and 160 mm Hg, who were either untreated or treated with a stable regimen of up to two antihypertensive therapies after the discontinuation of antihypertensive medication were enrolled into this study. Secondary forms of hypertension, orthostatic hypotension, a serum potassium concentration higher than 5 mmol/L, eGFR ≤ 30 mL/min/1.73 m^2^, type 1 diabetes, and poorly controlled type 2 diabetes (HbA1c > 9.0%) were exclusion criteria of the study. Overall, 394 patients were included in the study, of whom 377 underwent complete randomization. The study group included 93 black patients (24.7%) and 167 women (44.3%), and the mean age was 57 years. In this group, 302 patients received zilebesiran and 75 patients received the placebo. The subjects were assigned to one of 5 groups. Four of them received zilebesiran in various doses: these were 150, 300, or 600 mg once every 6 months or 300 mg once every 3 months, administered by subcutaneous administration. The fifth group consisted of patients receiving placebo once every 3 months. The observation period lasted 6 months. Ambulatory BP monitoring assessments were conducted using an automated device within a 24 h measurement period every 20 min during the day and every 30 min during the night. Measurements were completed during screening and in months 1, 3, and 6. Automated office BP measurements were performed during the screening period and on day 1, in week 2, and monthly thereafter through 6 months. At 3 months, 24 h mean ambulatory SBP decreased in the group with zilebesiran after treatment with 150 mg, 300 mg once every 3 months or every 6 months, or 600 mg. In the placebo group, ambulatory SBP increased (Figure 3). For 6 months, 60.9% of patients receiving zilebesiran had adverse events vs. 50.7% patients receiving placebo and 3.6% had serious adverse events vs. 6.7% receiving placebo. Adverse drug reactions were mild to moderate in severity. In the group of patients receiving zilebesiran, the most common (over 5% of patients) were local injection site reactions of erythema with accompanying pain (6.3% of reported adverse reactions) and hyperkalemia, defined in this study as a serum potassium concentration above 5.2 mmol/L (5.3% of reported adverse reactions). None of the cases of hyperkalemia led to the withdrawal of the patient from observation. In the placebo group, hyperkalemia occurred in 1% of the studied subjects, and no injection site reactions were observed [30].

The next stage of evaluating the effectiveness of zilebesiran was the phase 2, randomized, double-blind, and placebo-controlled study called KARDIA-2. It evaluated the efficacy, safety, and pharmacodynamics of zilebesiran added to standard antihypertensive therapy in patients with uncontrolled hypertension. The study enrolled adults with mild to moderate hypertension who were untreated (seated office systolic blood pressure 155–180 mm Hg) or receiving one or two antihypertensive drugs (seated office systolic blood pressure 145–180 mm Hg). During the run-in period, patients had their antihypertensive treatment modified: their previous medications were discontinued and they started oral antihypertensive monotherapy (indapamide, amlodipine or olmesartan), administered once a day. After a 4-week run-in period, patients with a mean SBP of 130–160 mm Hg in 24 h ambulatory monitoring were randomized to the zilebesiran or placebo group in addition to their background antihypertensive therapy. The study included 667 patients divided 1:1 to zilebesiran or placebo. The distribution of patients according to antihypertensive therapy was as follows: 127 patients received indapamide, 239 received amlodipine, and 301 received olmesartan. The mean age of the patients was 58.5 years, 56.8% were male, and 28.0% were black or African American. After three months of follow-up, zilebesiran significantly reduced 24 h mean ambulatory SBP and office SBP compared with the placebo as an add-on therapy to indapamide, amlodipine, or olmesartan (*p* < 0.05 for all comparisons). Ambulatory SBP measurements were as follows: −12.1 mm Hg (−16.5, −7.6; *p* < 0.001) with indapamide, −9.7 mm Hg (−12.9, −6.6; *p* < 0.001) with amlodipine, and −4.0 mm Hg (−7.6, −0.3; *p* = 0.036) with olmesartan compared to placebo. Office SBP measurements were as follows: −18.5 mm Hg (−22.8, −14.2; *p* < 0.001) with indapamide, −10.2 mm Hg (−13.5, −6.9; *p* < 0.001) with amlodipine, and −7.0 mm Hg (−10.4, −3.6; *p* < 0.001) with olmesartan compared to placebo. Additionally, a significant reduction in 24 h ambulatory SBP values was observed in the indapamide and amlodipine groups—the reductions in SBP were −11.0 mm Hg (−14.7, −7.3; *p* < 0.001) and −7.9 mm Hg (−10.6, −5.3; *p* < 0.001), respectively, compared to placebo. In the olmesartan group, the difference between the study group and placebo was 1.6 mm Hg (−4.4, 1.2; *p* = 0.26). In office measurements after 6 months, SBP was significantly lower in all study groups compared to placebo: −13.6 mm Hg (−16.9, −10.3; *p* < 0.001) in combination with indapamide, −8.6 mm Hg (−10.9, −6.3; *p* < 0.001) in combination with amlodipine, and −4.6 mm Hg (−6.8, −2.4; *p* < 0.001) in combination with olmesartan. Additionally, zilebesiran had a favorable safety and tolerability profile over the 6-month study period. No deaths or complications leading to exclusion from the study were reported. Episodes of hypotension were mild and resolved without intervention. In the first three months of follow-up, there was a tendency for hyperkalemia and increased serum creatinine concentrations, but these were resolved without intervention within two weeks. Hyperkalemia, an adverse event, was defined in this study as a serum potassium concentration above 5.5 mmol/L and required confirmation in a subsequent test. Hyperkalemia confirmed in two measurement was found in 1.6% of subjects in the indapamide group, in 1.7% of subjects in the amlodipine group, and in 1.3% of subjects in the olmesartan group. In comparison, in subjects receiving placebo, hyperkalemia did not occur. A decrease in eGFR by more than 30%, confirmed in two tests, was found in 4.8% of subjects in the indapamide group, in 0.8% of subjects in the amlodipine group, and in 2.7% of subjects in the olmesartan group. For comparison, in subjects receiving placebo, the aforementioned decrease in eGFR did not occur in the indapamide group but was observed in 1.7% of subjects in the amlodipine group and in 0.7% of subjects in the olmesartan group [31,32] (Table 1 and Table 2).

## 5. Zilebesiran—The Possible Clinical Aspects

Zilebesiran, used in doses of 150 mg and higher in patients with hypertension, has a long-term antihypertensive effect, effective for at least 6 months. In clinical studies described above, a reduction in blood pressure by over 10 mm Hg compared to is observed. Zilebesiran reduces blood pressure slowly. Therefore, it is not used in the treatment of hypertensive crises, but because of the gradual reduction in blood pressure, hypotensive episodes are not observed immediately after drug administration. Drug tolerance in the patients studied was good. The most common adverse effects were transient, local, mild inflammatory skin reaction at the injection site and hyperkalemia. It is worth noting, however, that hyperkalemia was mild, did not lead to termination of participation in the study, and involved 5% of the study participants [30,31]. Two important possible problems related to zilebesiran therapy should be more deeply discussed: risk of hyperkalemia and risk of transient hypotension during some specific situations, i.e., hypovolemia or infection.

Among patients treated with zilebesiran, hyperkalemia occurred in 5.3%, and it was mild and transient. Therefore, it seems that this risk is similar to the hyperkalemia risk related to ACEI or ARB therapy. Zilebesiran acts by silencing the AGT gene in hepatocytes, which leads to a significant reduction in the production of angiotensinogen. This results in the lack of activation of the entire RAAS cascade, a decrease in the concentration of angiotensin I and angiotensin II, and consequently a decrease in the secretion of aldosterone by the adrenal cortex. Physiologically, aldosterone, by binding to the mineralocorticoid receptor, increases the number and activity of sodium–potassium cotransporters in the cells of the distal tubules. At the same time, it increases sodium reabsorption and simultaneous potassium excretion. Reduction in aldosterone concentration leads to a decrease in sodium reabsorption and does not allow for the elimination of potassium via the aforementioned cotransporters [30]. Also, in the case of angiotensin-converting enzyme inhibitors (ACEIs) and angiotensin II receptor blockers (ARBs), one of the significant side effects is hyperkalemia. This phenomenon results directly from the mechanism of action of these drugs on the renin–angiotensin–aldosterone system. ARBs are antagonists of angiotensin II type 1 receptors; after binding to the receptor, they block it and reduce the tissue effect of angiotensin II. ACEIs, in turn, inhibit the action of the angiotensin-converting enzyme (ACE), which is responsible for the conversion of angiotensin I into angiotensin II, thereby reducing its serum concentration. Through the previously described mechanisms, they affect the reduction in angiotensin II concentration or the lack of its effect and, consequently, reduce the secretion of aldosterone by the adrenal cortex. As a result, there is a reduction in the reabsorption of sodium and water and a reduction in the excretion of potassium in the distal renal tubules. The incidence of hyperkalemia in the general population after the use of ACEIs or ARBs in numerous observations did not exceed 10%. Bandak et al., studying a group of 69,426 subjects with eGFR > 60 mL/min per 1.73 m^2^ who were taking ACEIs or ARBs, found a potassium serum concentration above 5 mmol/L in 5.6% of the study group, and a potassium serum concentration above 5.5 mmol/L in 1.7% of the study group [33]. Chang et al., in a group of 194,456 subjects during a three-year follow-up, found a potassium serum concentration above 5 mmol/L in 10.8% of the study group and a potassium serum concentration above 5.5 mmol/L in 2.3% of the study group [34]. Taking into account the analyzed thresholds of the serum potassium concentration, hyperkalemia after ACEI or ARB treatment is similarly to the hyperkalemia risk after zilebesiran therapy.

As mentioned, a single dose of small interfering RNA causes a significant reduction in angiotensinogen production in hepatocytes, and this effect lasts up to 6 months. Therefore, this significant angiotensinogen deficiency raises concerns in the context of the possible occurrence of acute clinical situations associated with hypotension, e.g., hypovolemia. In these situations, the increase and maintenance of perfusion pressure is directly dependent on the increased production of angiotensinogen, which is long-term blocked in the case of siRNA. In the face of such a situation, other possibilities of increasing blood pressure in hypotension in the case of angiotensinogen deficiency were sought. Uijl et al. [26] analyzed the possibility of using conventional vasoconstrictor drugs for this purpose. In the study, they used spontaneously hypertensive rats with implanted telemetry transmitters for continuous measurement of blood pressure and heart rate. A low-salt diet was used to reduce blood pressure in all rats. On days 7 and 21 of observation, all rats received angiotensinogen siRNA by subcutaneous injection. The mean arterial pressure (MAP), which was 136 mm Hg ± 3 mm Hg at baseline, was reduced to 130 mm Hg ± 2 mm Hg after the low-salt diet (*p* < 0.0001). After adding angiotensinogen siRNA to the already used low-salt diet, the mean arterial pressure was reduced to 117 mm Hg ± 2 mm Hg (*p* < 0.0001). The plasma angiotensinogen concentration decreased by 99.2 ± 0.1% after siRNA administration. Before and during siRNA treatment, angiotensinogen II and norepinephrine were administered by intravenous injection to assess the pressor response. The siRNA-induced blood pressure reduction was reversible by the intravenous administration of angiotensin II or norepinephrine. In the case of angiotensin II, the efficacy in increasing MAP during siRNA treatment was greater, most likely due to the increased sensitivity of angiotensin II receptors in the situation of chronic reduction in angiotensin II concentration during siRNA treatment. Rats were then divided into three groups: those receiving fludrocortisone at a dose of 6 mg/kg body weight by subcutaneous injection (n = 7), those receiving midodrine at a dose of 4 mg/kg body weight by subcutaneous injection followed by 8 mg/kg body weight orally (n = 6), and a group with a diet changed from low- to high-salt. The subcutaneous administration of fludrocortisone caused a significant increase in MAP on the fifth day of treatment (*p* < 0.05) and a return to the baseline MAP values on the seventh day. In the group of rats treated with midodrine, no increase in blood pressure was observed after either subcutaneous or double oral administration. Increasing dietary sodium intake increased MAP by 8 mm Hg ± 0.4 mm Hg on the first day of treatment (*p* < 0.0001). MAP returned to baseline by the fourth day. In summary, this study showed that the decrease in blood pressure using angiotensinogen siRNA can be reduced by using vasopressors. In an emergency situation, noradrenaline will be effective, while in more chronic therapy, a high-salt diet, or cases where its sole use to treat patients on a high-salt diet are ineffective, treatment with fludrocortisone might be effective. The above results are important in the clinical context, because a significant decrease in angiotensinogen synthesis after using siRNA may result in a lack of increase in RAAS activity in the case of hypovolemia [35].

Another, very interesting alternative to classical vasopressors in the case of a sudden drop in blood pressure in patients using siRNA of angiotensinogen is the technology of reverse siRNA silencing called REVERSIR. REVERSIR technology consists of the supply of synthetic short, single-stranded oligonucleotides complementary to the antisense siRNA strand connected to the RISC. Similarly to angiotensinogen siRNA, the aforementioned oligonucleotides are administered and transported in the same way: in a complex with GalNAc, they are transported specifically to the receptors for N-acetylgalactosamine present on the hepatocytes cell membrane and undergo endocytosis into the cells. RVR oligonucleotides bind with high affinity to the antisense siRNA strand in the RISC, which blocks the recognition and silencing of the corresponding angiotensinogen mRNA and allows its production to resume. Ye et al. evaluated the efficacy of REVERSIR in spontaneously hypertensive rats using angiotensinogen siRNA [36]. Thirty-two spontaneously hypertensive rats were given a subcutaneous injection of AGT siRNA at a dose of 10 mg/kg body weight. After 3 weeks, 16 rats from this group received REVERSIR at three different doses (1, 10, or 20 mg/kg body weight). One week after REVERSIR administration, 8 rats from this group were given another dose of AGT siRNA to evaluate its efficacy after REVERSIR. The baseline MAP in the whole group was 144 mmHg ±1 mm Hg. After the first dose of AGT siRNA, MAP decreased by about 16 mm Hg, and the concentration of angiotensinogen decreased by 95%. In the next phase of the study, all doses of REVERSIR restored MAP to baseline values within 4 to 7 days. However, it is worth noting that doses of 10 mg/kg body weight and 20 mg/kg body weight restored angiotensinogen concentration to baseline values, while 1 mg/kg body weight increased angiotensinogen concentration to about 50% of baseline values. After the second dose of AGT siRNA in the group that received 1 mg/kg body weight, REVERSIR MAP decreased to the same extent as after the initial dose. In the group that received 10 mg/kg body, weight REVERSIR MAP decreased by only about 8 mm Hg [36].

Despite the described efficacy of treatment, there are still many key questions regarding the effect of zilebesiran treatment. Proper blood pressure control is one of the most important modifiable risk factors for cardiovascular disease. In this case, one of the key issues is the reduction in the frequency of cardiovascular disease complications and mortality in patients with hypertension treated with zilebesiran and the possible improvement of prognosis in heart failure patients using this drug. Due to the fact that good control of blood pressure also contributes to slowing down the development and progression of chronic kidney disease, the potential nephroprotective effect is also worth assessing. The results of long-term observations of patients treated with zilebesiran and the assessment of its effect after a longer period of use or the duration of this effect after a single administration are also interesting. Further research is needed to address long-term safety issues, as the observations to date are still relatively short. The use of zilebesiran in special patient populations, such as the elderly, is also an interesting and necessary consideration, particularly in terms of its safety.

Due to the transient, low-titer appearance of antibodies against zilebesiran in 2.5% of the study subjects in the phase 1 study, the possibility of developing clinically significant resistance to long-term treatment with this drug is also interesting, as already described [5].

Some questions may be answered by the results of the KARDIA-3 study, which began in April 2024 and is still ongoing. It aims to assess the efficacy and safety of zilebesiran as an adjunct therapy in patients with diagnosed cardiovascular disease or high cardiovascular risk, with or without advanced chronic kidney disease, and uncontrolled hypertension despite stable treatment with two or four standard antihypertensive drugs.

## Figures and Tables

**Table 1:** Zilebesiran—comparison of clinical studies.

	Patient Populations	Study Design	Results
Desai et al. phase I [29]	Aged 18 to 65 years, Treated or untreated hypertension—mean SBP ≥ 130 mm Hg, n = 107	Four parts: single ascending dose (10, 25, 50, 100, 200, 400, or 800 mg) single dose 800 mg + low-salt diet single dose 800 mg + high-salt diet single dose 800 mg with concomitant irbesartan	Doses ≥ 100 mg reduced serum AGT concentrations ≥ 90%, Single doses ≥ 200 mg: decreases in SBP > 10 mm Hg and DBP > 5 mm Hg) through week 8
KARDIA-1 phase II [30]	Aged 18 to 75 years, Moderate hypertension—SBP 135–160 mm Hg, n = 394	Five groups: zilebesiran in various doses: 150, 300, or 600 mg once every 6 months or 300 mg once every 3 months, placebo once every 3 months by subcutaneous administration	Decrease in SBP: - 7.3 mm Hg for 150 mg every 6 months, - 10.0 mm Hg for 300 mg every 6 months, - 10.0 mm Hg for 300 mg every 3 months, - 8.9 mm Hg for 600 mg every 6 months
KARDIA-2 phase II [31]	Adults with mild to moderate hypertension who were untreated (SBP 155–180 mm Hg) or receiving one or two antihypertensive drugs (SBP 145–180 mm Hg), n = 667	Three groups: Indapamide 2.5 mg daily, amlodipine 5 mg daily, or olmesartan 40 mg daily After 4 weeks in every of three groups: randomized double-blind 1:1 to zilebesiran 600 mg s.c. or Placebo subgroups	Reduced serum AGT concentrations ≥ 95%, Decrease in office SBP in zilebesiran’s groups: - 18.5 mm Hg with indapamide, - 10.2 mm Hg with amlodipine - 7.0 mm Hg with olmesartan

**Table 2:** Zilebesiran—side effects comparison in clinical trials.

	Injection Site Reactions	Hyperkalemia	Hypotension	Worsening Renal Function	Immunogenicity
Desai et al. phase I [29]	9%	0	0	0	2%
KARDIA-1 phase II [30]	6%	5%	1%	0	-
KARDIA-2 phase II [31]	3%	6%	4%	1%	-

## Data Availability

No new data were created or analyzed in this study.
